# D-cycloserine improves synaptic transmission in an animal mode of Rett syndrome

**DOI:** 10.1371/journal.pone.0183026

**Published:** 2017-08-16

**Authors:** Elisa S. Na, Héctor De Jesús-Cortés, Arlene Martinez-Rivera, Zeeba D. Kabir, Jieqi Wang, Vijayashree Ramesh, Yasemin Onder, Anjali M. Rajadhyaksha, Lisa M. Monteggia, Andrew A. Pieper

**Affiliations:** 1 Department of Psychology & Philosophy, Texas Woman’s University, Denton, TX, United States of America; 2 Picower Institute for Learning and Memory, Massachusetts Institute of Technology, Cambridge, MA, United States of America; 3 Division of Pediatric Neurology, Department of Pediatrics, Weill Cornell Medicine, Cornell University, New York, NY, United States of America; 4 Weill Cornell Autism Research Program, Weill Cornell Medicine, Cornell University, New York, NY, United States of America; 5 Department of Psychiatry, University of Iowa Carver College of Medicine, Iowa City, IA, United States of America; 6 Department of Neuroscience, UT Southwestern Medical Center, Dallas, TX, United States of America; 7 Feil Family Brain and Mind Research Institute, Weill Cornell Medicine, Cornell University, New York, NY, United States of America; 8 Department of Neurology, University of Iowa Carver College of Medicine, Iowa City, IA, United States of America; 9 Department of Free Radical and Radiation Biology, University of Iowa Carver College of Medicine, Iowa City, IA, United States of America; 10 Department of Veterans Affairs, University of Iowa Carver College of Medicine, Iowa City, IA, United States of America; 11 Pappajohn Biomedical Institute, University of Iowa Carver College of Medicine, Iowa City, IA, United States of America; Consiglio Nazionale delle Ricerche, ITALY

## Abstract

Rett syndrome (RTT), a leading cause of intellectual disability in girls, is predominantly caused by mutations in the X-linked gene *MECP2*. Disruption of *Mecp2* in mice recapitulates major features of RTT, including neurobehavioral abnormalities, which can be reversed by re-expression of normal *Mecp2*. Thus, there is reason to believe that RTT could be amenable to therapeutic intervention throughout the lifespan of patients after the onset of symptoms. A common feature underlying neuropsychiatric disorders, including RTT, is altered synaptic function in the brain. Here, we show that *Mecp2*^*tm1*.*1Jae/y*^ mice display lower presynaptic function as assessed by paired pulse ratio, as well as decreased long term potentiation (LTP) at hippocampal Schaffer–collateral-CA1 synapses. Treatment of *Mecp2*^*tm1*.*1Jae/y*^ mice with D-cycloserine (DCS), an FDA-approved analog of the amino acid D-alanine with antibiotic and glycinergic activity, corrected the presynaptic but not LTP deficit without affecting deficient hippocampal BDNF levels. DCS treatment did, however, partially restore lower BDNF levels in the brain stem and striatum. Thus, treatment with DCS may mitigate the severity of some of the neurobehavioral symptoms experienced by patients with Rett syndrome.

## Introduction

Rett syndrome (RTT) causes intellectual disability [1,2] in 1 out of every 10,000 females worldwide, and is caused ~95% of the time by mutations in the X chromosomal gene *MECP2* that encodes for methyl-CpG-binding protein 2 (MeCP2) [3,4]. MeCP2 is a highly-abundant DNA binding protein that affects expression of multiple downstream genes and signaling pathways [5]. Most disease-causing mutations arise in the male germ line, resulting in almost all RTT patients being female heterozygotes with one normal and one mutated copy of *Mecp2*. Girls with RTT are born normally after full term pregnancy without perinatal complications, but from 6–18 months of age they experience regression that precludes attainment of normal developmental milestones and is associated with reduced head growth, seizures, intellectual disability, regressed motor function, emotional lability, autonomic dysregulation, anxiety, scoliosis, and potentially fatal aberrant respiratory function [6–8]. There is currently no effective treatment for patients with RTT.

Disruption of *Mecp2* in mice recapitulates major features of RTT [9–13], and re-expression of normal *Mecp2* reverses the deficits seen in mice [14]. In addition, post-mortem studies of brains of patients with RTT have demonstrated lack of neuronal cell loss or neurodegeneration, indicating that prolonged loss of MeCP2 protein does not result in major changes in brain structure. Thus, there is reason to believe that treatment of patients throughout their lifespan, well after the onset of developmental regression, could mitigate the severity of symptoms associated with RTT. Previously, aberrant synaptic neurotransmission thought to be related to the cognitive and functional deficits underlying RTT has been demonstrated in the brains of behaviorally-symptomatic *Mecp2*^*tm1*.*1Jae/y*^ mice. Specifically, *Mecp2*^*tm1*.*1Jae/y*^ mice display impaired excitatory glutamatergic neurotransmission, as indicated by deficient hippocampal long-term potentiation (LTP) associated with altered expression of N-methyl-D-aspartate (NMDA) receptor subunits [15]. Presynaptic function is also disrupted in *Mecp2*^*tm1*.*1Jae/y*^ mice, as evidenced by significantly reduced paired-pulse facilitation in the brain [15,16]. Notably, altered patterns of expression of both the NMDA- and αgamma-amino-3-hydroxy-5-methyl-4-isoxazolepropionic acid (AMPA)-type glutamate receptors have been observed in postmortem brain samples of patients with RTT [17,18], as well as elevated CSF glutamate levels in patients with RTT compared to subjects with other forms of autism-spectrum disorder [19,20]. These findings further support a role for dysfunctional glutamatergic signaling in RTT, suggesting possible therapeutic potential for pharmacologic modulation of this system in patients.

D-cycloserine (DCS) is an amino acid derivative sold under the brand name Seromycin. In addition to antibiotic efficacy against extensively drug-resistant strains of *Mycobacterium tuberculosis*, DCS is a partial agonist at the strychnine-insensitive glycine recognition site on the NR1 subunit of NMDA receptors in the brain. In this capacity, DCS increases calcium influx in response to NMDA-mediated glutamatergic signaling, without causing neurotoxicity [21]. In animal models, DCS enhances extinction of conditioned fear [22–27], a form of learning that represents a valid preclinical model of exposure-based therapy that is dependent on NMDA receptors for people with anxiety disorders [28–32]-. Treatment with DCS also shows efficacy in preclinical models of deficient fear extinction after stress [33,34], alcohol withdrawal [35], and REM sleep deprivation [36], as well as in mutant mice harboring the brain-derived neurotrophic factor (BDNF) Val66Met polymorphism associated with increased susceptibility to neuropsychiatric disease [37]. In humans, treatment with DCS enhances the efficacy of exposure therapy for various forms of maladaptive fear, including acrophobia [38], social anxiety [39,40], obsessive-compulsive disorder [41, 42], and panic disorder [43]. Because deficits in neurocognitive functioning are a prominent feature in patients with Rett, we applied the *Mecp2*^*tm1*.*1Jae/y*^ mouse model of RTT to determine whether DCS might mitigate synaptic deficits associated with this condition.

## Materials and methods

### Animals

All animal procedures were performed in accordance with UT Southwestern Medical Center institutional animal care and use committee’s regulations. Male mice were housed in temperature controlled conditions, provided food and water *ad libitum*, and maintained on a 12-hr light/dark cycle (6 A.M. to 6 P.M.). The *Mecp2*^*tm1*.*1Jae/y*^ mouse colony [44] was maintained on C57Bl/6J background. Animal protocols were approved by the ethics committees at both Ut Southwestern Medical Center and Weill Cornell Medicine.

### D-cycloserine (DCS) administration

DCS was obtained from Sigma-Aldrich (C6880 SIGMA), dissolved in PBS at pH 7.4, and administered by intraperitoneal injection to animals at 20 mg/kg/day every day beginning at 3 weeks of age and continuing until death. Injection sites were alternated left and right every other day. WT and *Mecp2*^*tm1*.*1Jae/y*^ mice were randomly assigned to either vehicle or DCS treatment, and subsequently coded unrelated to genotype or treatment group in order to blind experimenters to group conditions.

### Behavioral analysis

Analysis was conducted blind to genotype and treatment at 18 weeks of age, according to established protocols [12].

#### Locomotor activity analysis

Locomotor activity was determined for 8 hours during the active phase of mice by automated counting of the number of beam breaks in an 18 cM wide X 12 cM high open field.

#### Apnea analysis

Apnea measurements were determined by placing mice in a whole body plethysmograph (Buxco) to measure breathing in an unrestrained state. Mice were acclimated for one half hour in the chamber before apneas were recorded for a 15 minute period. Apneas were defined as a pause in the breathing cycle lasting longer than 1.5 seconds.

#### Grip strength analysis

Grip strength was determined in mice suspended by their forelimbs on a standard 1 cm diameter bar positioned 20 cm above standard cage bedding, and time to fall was recorded.

#### Tremor analysis

Tremor score was determined through a 3 point observational scoring system while the mouse was standing on the flat palm of the observer’s hand. A score of 0 indicated no tremor, 1 indicated mild intermittent tremor, and 2 indicated constant tremor or intermittent severe tremor.

#### Walking gait analysis

Walking gait score was determined through a 3 point observational scoring system when the mouse was allowed to ambulate on a flat table top. A score of 0 indicated normal gait, 1 indicated hind legs spread wider than normal when ambulating, 2 indicated severe abnormality either with tremor when feet were lifted or with backwards walking by lifting both rear feet simultaneously.

#### Neurological function analysis

Neurological Function (NF) score was determined by summing the scores of general condition, hindlimb clasping, gait, and tremor. General condition score was determined by means of a 3 point observational scoring system that indicated coat condition, appearance of eyes, and body stance. A score of 0 meant clean shiny coat, clear eyes and normal stance. A score of 1 indicated dull coat or poorly-groomed appearance, dull eyes, and mildly hunched stance. A score of 2 indicated piloerection, crusted or narrowed eyes, and fully hunched stance. Hindlimb clasping was defined as when the hindlimbs were spread in the area less than their body width while the animal was suspended by its tail. No clasping received a score of 0, and clasping received a score of 1.

### Hippocampal slice electrophysiology

18 week old mice that had been treated since 3 weeks of age with either vehicle or DCS (20 mg/kg/day IP), and not exposed to any behavioral testing, were used for electrophysiologic studies. The experimenter was blind to genotype and treatment during the recording and data analysis stages. Mice were anesthetized with Euthasol (30 mg/ml, 0.2 ml i.p.) before decapitation. Brains were removed and immersed for 2–3 minutes in ice-cold artificial cerebral spinal fluid (aCSF) containing (in mM): 119 NaCl, 2.5 KCl, 2.5 CaCl_2_, 1.3 MgSO_4_, 1 NaH_2_PO_4_, 26 NaHCO_3_ and 10 glucose, continuously bubbled with 95% O_2_ and 5% CO_2_, pH 7.4. The hippocampi were extracted and cut with a vibratome into 350 μm thick transverse sections in ice cold ACSF. Sections recovered in oxygenated ACSF for at least 1 hour at 32°C. Hippocampal slices were transferred into a recording chamber and superfused with ACSF at a constant rate of 2.5 ml/minutes at 30°C. Field excitatory post synaptic (fEPSP) potentials were recorded with glass recording electrodes filled with aCSF (Sutter Instruments, resistance, 1–2 MΩ). Extracellular stimuli were delivered by placing a bipolar platinum-tungsten stimulating electrode at the region of interest (A-M Systems Isolated Pulse Stimulator, Model 2100). The stimulating electrode was inserted to stimulate fibers of the Schaffer collateral pathway and the recording electrode was inserted into the CA1 just beneath the molecular layer. The stimulating and recording electrodes were separated by a distance of 300–350 μm. Electrical signals were amplified (A-M systems AC amplifier Model 1800), digitized, and stored on a PC for subsequent analysis using Labview 8.6 software (National Instruments). Input-output (I/O) relationship was determined by providing an ascending series of stimulus input intensities (range 4–24 μA) until the maximum amplitude response was reached. An input stimulus intensity that induces 40–60% of the maximum response was used for measuring paired-pulse ratios (PPR) and long-term potentiation (LTP). PPR was measured by giving 2 pulses at decreasing interstimulus intervals (500, 400, 200, 100, 50, 30, 20 ms) and analyzed by dividing the fEPSP slope of pulse 2 by pulse 1. Following 20 minutes of stable baseline, LTP was induced by high frequency stimulation using an input stimulus intensity that produces the maximum response (HFS, 2 100 Hz trains with 100 pulses with an interburst interval of 20 seconds). Slices that did not have 20 minutes of stable baseline were excluded from LTP analyses.

### BDNF ELISA

Animals started treatment of either vehicle or DCS at 3 weeks old and it continued for 4 weeks. Euthanasia was performed by anesthesia overdose followed by microdissection of hippocampus, striatum and brainstem and immediate flash freeze in liquid nitrogen. Total BDNF (tBDNF) protein level was measured using the BDNF Emax ImmunoAssay (ELISA) system (Promega), with recombinant mature BDNF as a standard and samples were performed in duplicate, with each group containing 10–14 samples. Protein was extracted and quantified following the manufacturer’s protocol. Tissue samples were homogenized in lysis buffer (150 mM NaCl, 1% Triton X-100, 25 mM HEPES, 2 mM NaF) containing phosphatase and protease inhibitors, and then incubated by rotation at 4°C for 1 h. Homogenized tissue was centrifuged at maximum speed and the supernatant containing total protein was collected and quantified using the BCA protein assay kit (Thermo Fisher Scientific). Each sample was diluted 1:1 with block and sample buffer (BSB), and placed in designated wells of a 96-well plate previously coated with BDNF antibody in carbonate buffer (25 mM Na2CO3 and 25 mM Na2HCO3, pH 9.7, incubated at 4°C), followed by blocking with BSB. A second coating of primary anti-human BDNF antibody was added, followed by horseradish peroxidase-conjugated secondary antibody. The colorimetric reaction was initiated by tetramethylbenzidine. After 10 min, the reaction was stopped by addition of 1N HCl, and absorbance was read at 450 nm on a plate reader (iMark Absorbance Microplate Reader, Bio-Rad Laboratories).

### BDNF western blot analysis

Forty micrograms of total protein lysate was separated on a 15% SDS protein gel with a Kaleidoscope-prestained protein standard (Bio-Rad). Blots were blocked in 5% non-fat dry milk and incubated in primary antibody (mature BDNF, Abcam, Catalog #ab108319 used at 1:5000 and secondary at 1:5000; proBDNF, Santa-Cruz Biotechnology, Catalog #sc-65514 used at 1:500 and secondary at 1:5000) for 24 hours at 4°degrees celsius. Blots were incubated in horseradish peroxidase-linked IgG-conjugated secondary antibody for 1 hour. Protein bands were visualized by chemiluminescence solution (Western Lightening, Perkin Elmer Life Sciences). Band at 15kDa was quantitated for mature BDNF, and band at 28kDa was quantitated for proBDNF. GAPDH (37 kDa; Abcam, Catalog# Ab22555 used at 1:20000 and secondary at 1:30000) was used as a loading control.

#### Statistics

For I/O relationships, slopes for each slice were calculated and a two way ANOVA was used to assess differences between groups. For PPR, paired pulse ratios were analyzed using a one-way ANOVA. If the overall F value was significant, Student Newman-Keuls post-hoc tests were used to determine significant differences between groups. LTP was analyzed using two way repeated measures ANOVA between vehicle groups. If the interaction effect was significant, Student Newman-Keuls was used as a post-hoc test. For the BDNF experiments, a two-way ANOVA was used followed by Bonferroni post-hoc test for factors with a main effect. Power analyses were conducted on PPR data to determine whether sample sizes were appropriate to achieve sufficient statistical power. Using GPower (Franz Faul, Universitat Kiel, Germany), we determined that the n numbers for the PPR experiments were sufficient to reject the null hypothesis, Power≥0.80 for 20, 30, and 100 ms interstimulus intervals and 0.7 for the 200 ms interstimulus interval.

## Results and discussion

### Treatment with DCS is safely tolerated by Mecp2^tm1.1Jae/y^ mice

**Fig 1:**
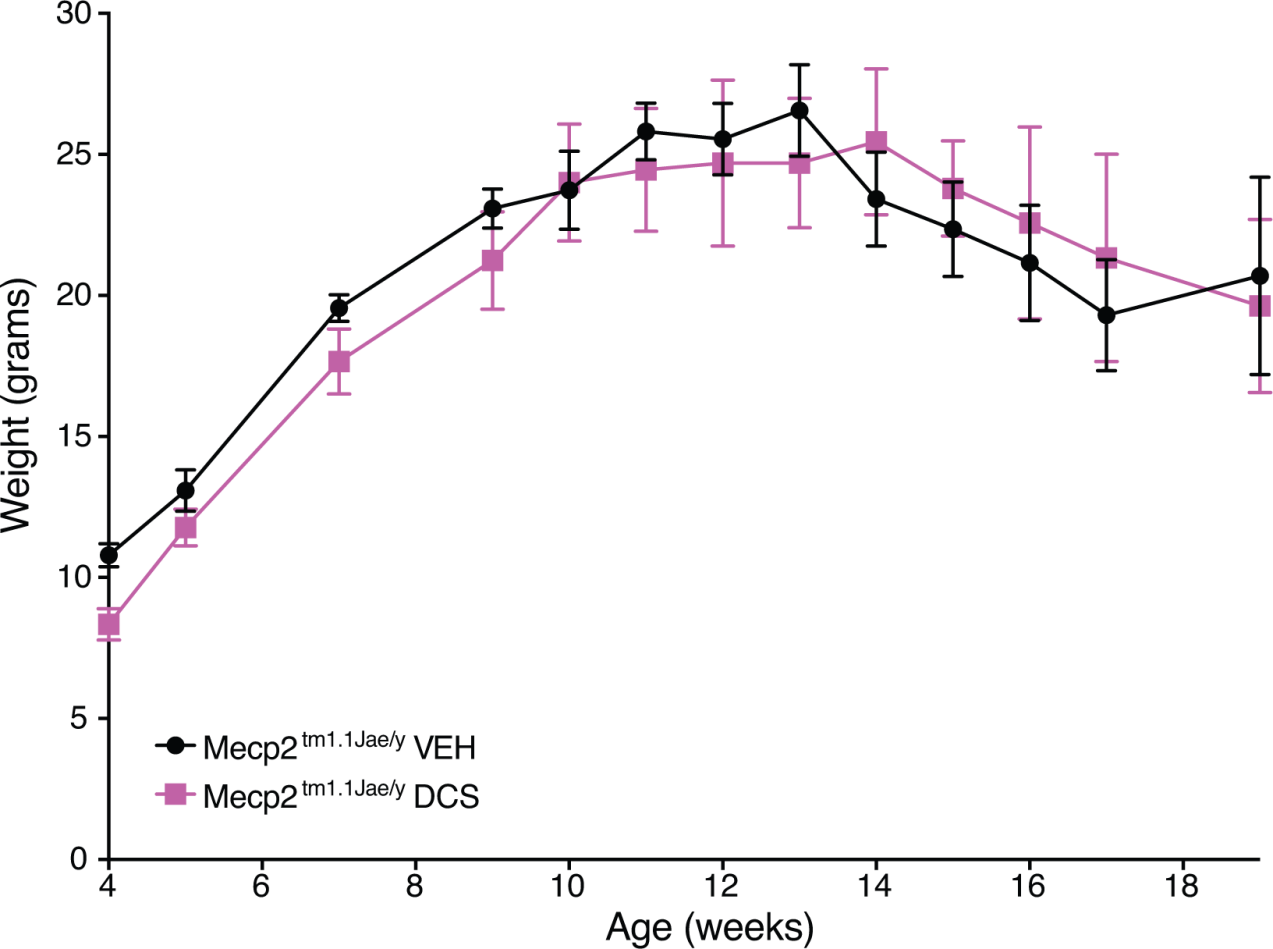
Administration of DCS (20 mg/kg/day IP) to *Mecp2*^*tm1*.*1Jae/y*^ mice did not alter body weight compared to *Mecp2*^*tm1*.*1Jae/y*^ mice receiving vehicle (VEH) injection (n = 5 per group). Mice were injected daily beginning at 3 weeks of age and continued throughout the life of the animal.

**Fig 2:**
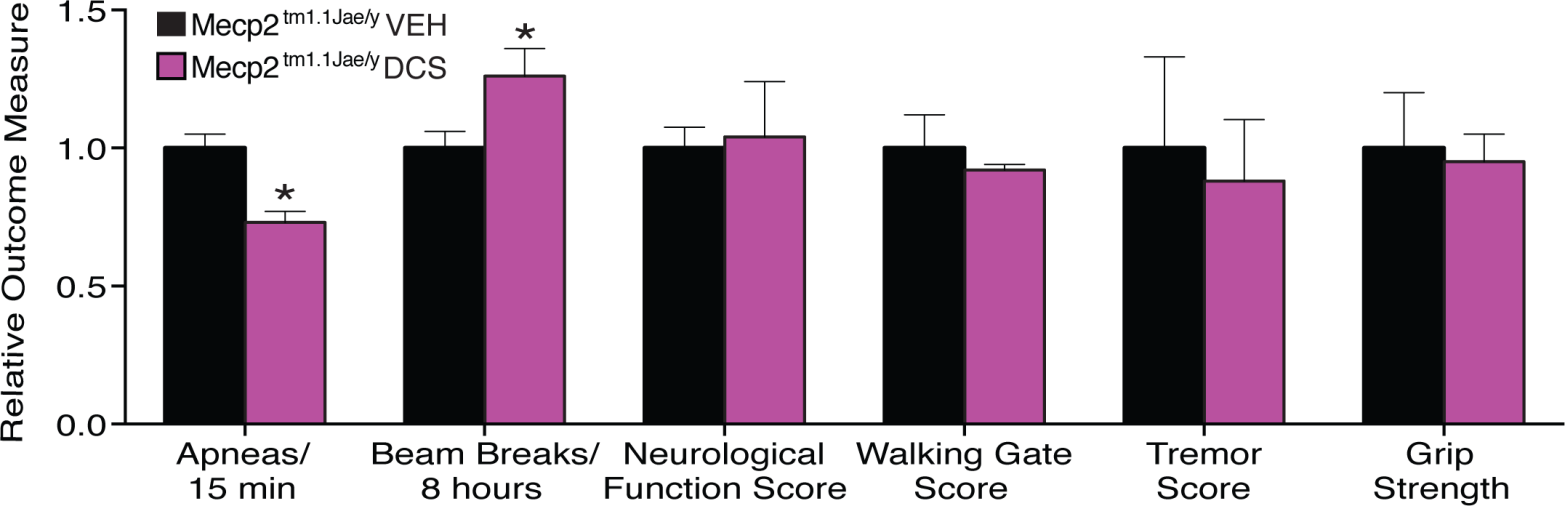
Administration of DCS (20 mg/kg/day IP) to *Mecp2*^*tm1*.*1Jae/y*^ mice, beginning at 3 weeks of age, improved the breathing pattern and spontaneous locomotor activity, but did not affect neurological function, walking gate, tremors or grip strength compared to *Mecp2*^*tm1*.*1Jae/y*^ mice receiving vehicle (VEH) injection (n = 5 per group), at 18 weeks of age.

Before conducting electrophysiologic testing of the hypothesis that DCS might help treat synaptic deficits related to Rett syndrome, we examined whether *Mecp2*^*tm1*.*1Jae/y*^ mice, a well-characterized animal model of RTT, would display obvious signs of detriment with chronic DCS exposure. Treatment with DCS (20 mg/kg/day) or vehicle was initiated in 3 week old *Mecp2*^*tm1*.*1Jae/y*^ mice and continued every day until experimentation. This did not affect weight gain of *Mecp2*^*tm1*.*1Jae/y*^ mice (Fig 1), indicating no obvious toxicity of the drug. Encouragingly, this treatment regimen moderately reduced breathing apneas in 18 week old *Mecp2*^*tm1*.*1Jae/y*^ mice by 20% (t(8) = 4.55, p = .002), and also improved basal locomotor activity by about 20% (t(8) = 2.76, p = .025) (Fig 2). Vehicle-treated *Mecp2*^*tm1*.*1Jae/y*^ mice showed the expected abnormalities in these parameters (Fig 2), as the raw numbers for these modalities were compared to previous published data from our laboratory and found to be comparable across all measures [12]. No changes were observed in overall neurologic function, walking gait, tremor, or grip strength between DCS- and vehicle-treated mice (Fig 2). Treatment with DCS also resulted in a modest enhancement of the 50% survival rate of *Mecp2*^*tm1*.*1Jae/y*^ mice to 18.5 weeks, compared to 13.5 weeks in the vehicle-treated group (p = 0.0826, Gehan-Breslow-Wilcoxon test). Taken together, these results indicate that chronic treatment with DCS is not harmful to *Mecp2*^*tm1*.*1Jae/y*^ mice, and in fact may possibly offer some measure of neurobehavioral benefit. More rigorously highly powered studies in the future will be needed to determine whether the behavioral benefits suggested here in our safety screen in fact manifest prominently in this model as a function of treatment with DCS.

### Treatment of Mecp2^tm1.1Jae/y^ mice with DCS restores normal hippocampal paired-pulse ratio

**Fig 3:**
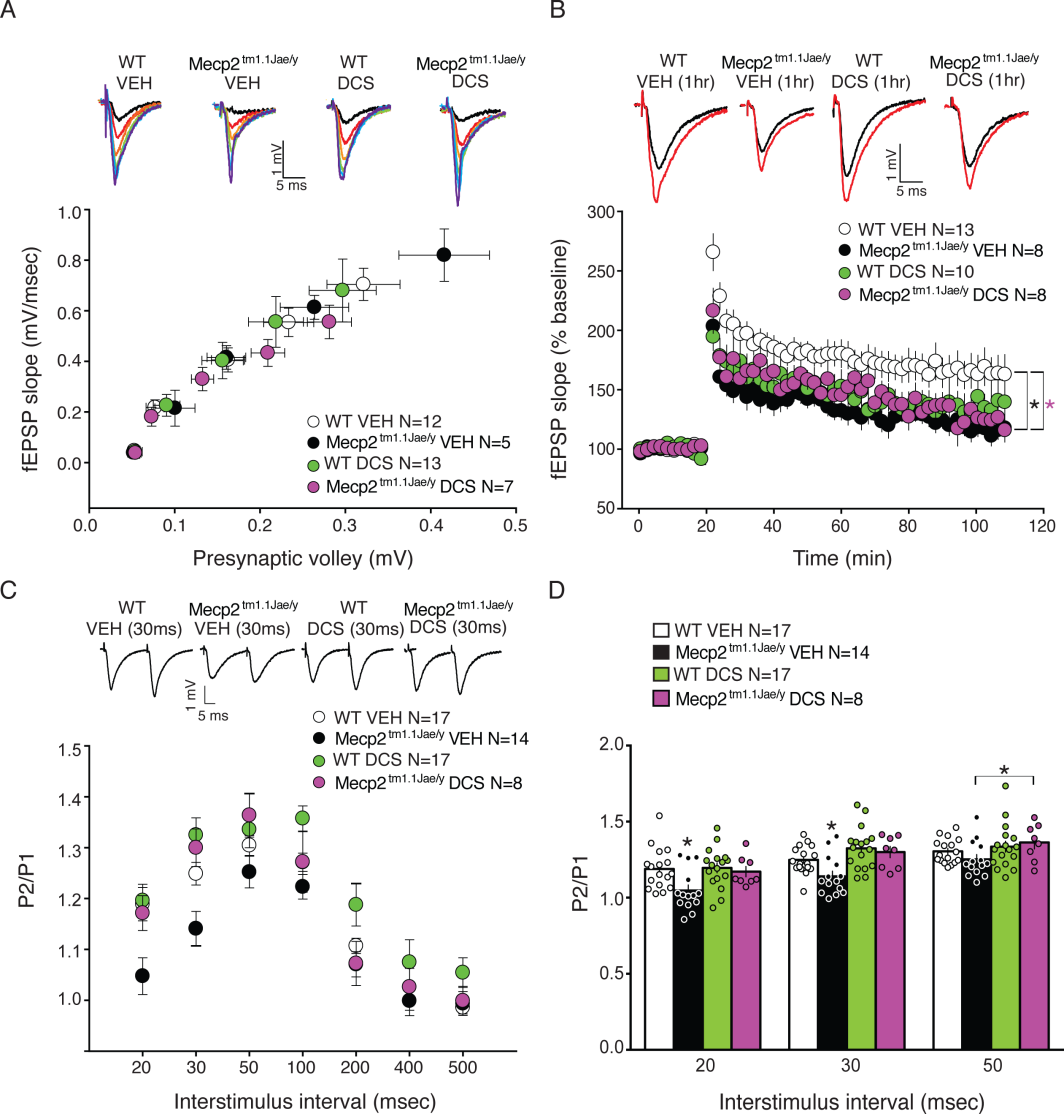
PPR deficits but not LTP are ameliorated in *Mecp2*^*tm1*.*1Jae/y*^ mice given chronic D-cycloserine treatment (20 mg/kg/day IP). A, There were no significant differences in input output slopes in *Mecp2*^*tm1*.*1Jae/y*^ and WT mice given vehicle (VEH) or drug, indicating no change in gross number of active synapses relative to WT VEH mice. B, LTP is substantially reduced in *Mecp2*^*tm1*.*1Jae/y*^ mice compared to WT VEH controls. D-cycloserine had no effect on *Mecp2*^*tm1*.*1Jae/y*^ mice compared to *Mecp2*^*tm1*.*1Jae/y*^ mice given VEH. D-cycloserine significantly attenuated LTP responses in WT mice compared to WT VEH mice. C. PPR experiment. All groups are shown. D. Bar graph demonstrating that chronic D-cycloserine treatment reversed paired pulse ratios at 20, 30, and 50 msec interstimulus intervals in the *Mecp2*^*tm1*.*1Jae/y*^ group compared to *Mecp2*^*tm1*.*1Jae/y*^ mice given VEH treatment suggesting that D-cycloserine ameliorates PPR deficits at these intervals. *Mecp2*^*tm1*.*1Jae/y*^ VEH group had significantly lower PPR compared to all groups at the 20 and 30 msec intervals. An independent t-test revealed a significant difference between the *Mecp2*^*tm1*.*1Jae/y*^ VEH and *Mecp2*^*tm1*.*1Jae/y*^ D-cycloserine groups at the 50 msec interstimulus interval. * p≤.05. Representative sample traces from each group are shown in top of respective graph.

To examine electrophysiologic measures of synaptic function, *ex vivo* analysis was conducted on acute hippocampal brain slices derived from 18 week old behaviorally-naïve vehicle- and DCS-treated *Mecp2*^*tm1*.*1Jae/y*^ and wild type littermate mice. I/O curves were generated by placing a stimulating electrode in the Schaffer collaterals and recording field potentials in the hippocampal CA1 region. The I/O slopes of presynaptic volley amplitude to the fEPSC slope were not significantly different between any of the four groups, suggesting that DCS treatment did not affect basal synaptic transmission in mice (Fig 3A).

To assess LTP, we stimulated the Schaffer-collateral pathway using high frequency stimulation (2 trains of enhanced pulses 100 Hz), a stimulation paradigm previously shown to induce LTP in a *Mecp2* overexpression mouse line [45]. With respect to LTP, two-way repeated measures ANOVA revealed significant differences between vehicle-treated *Mecp2*^*tm1*.*1Jae/y*^ and WT mice, demonstrating significant impairments in high frequency stimulation-induced LTP in hippocampal slices from *Mecp2*^*tm1*.*1Jae/y*^ mice. A significant interaction effect was seen between vehicle groups (F(54, 1154) = 4.2, p = .001). Post-hoc analyses revealed a significant attenuation in LTP in *Mecp2*^*tm1*.*1Jae/y*^ mice. A significant interaction effect was also seen between WT groups (F(54,1264) = 2.4, p = .001), and post-hoc analyses showed a significant attenuation in LTP in DCS-treated WT slices 35% of the time after HFS stimulation, compared to vehicle-treated WT hippocampal slices. In *Mecp2*^*tm1*.*1Jae/y*^ mice, we did not observe any significant difference in LTP due to DCS compared to vehicle treatment (Fig 3B).

We next examined PPR, an index of presynaptic probability of release, by recording the response of two pulses separated by varying interstimulus intervals and then comparing the second and first responses. PPR was significantly different at 20 and 30 msec interstimulus intervals, with F(3,55) = 5.03, p = .004 and F(3,55) = 6.7, p = .001, respectively, with DCS-treatment reversing PPR deficits in *Mecp2*^*tm1*.*1Jae/y*^ hippocampal slices (Fig 3C and 3D). Post-hoc analyses revealed differences between vehicle-treated *Mecp2*^*tm1*.*1Jae/y*^ hippocampal slices and all other groups (p<0.05). We also included an effect size analysis using Cohen’s d and discovered that our Cohen’s d values were -1.1 for the 20 msec interstimulus interval (ISI) and -1.4 for the 30 msec ISI when comparing the effect size between the vehicle-treated *Mecp2*^*tm1*.*1Jae/y*^ and DCS-treated *Mecp2*^*tm1*.*1Jae/y*^ mice, indicating that over 80% of our DCS-treated *Mecp2*^*tm1*.*1Jae/y*^ mice will be above the mean of the vehicle-treated *Mecp2*^*tm1*.*1Jae/y*^ group. PPR was not statistically significant at 50 msec, but vehicle- and DCS-treated *Mecp2*^*tm1*.*1Jae/y*^ slices showed significantly different PPRs, as determined by an independent t-test (2-tailed), t(20) = 2.1, p = .05. PPR was also significantly different between vehicle- and DCS-treated WT hippocampal slices at the 100 msec interstimulus interval (F(3,55) = 4.6, p = .006; Student-Newman-Keuls, p = .05). DCS-treated WT hippocampal slices showed a higher PPR at the 500 msec interstimulus interval compared to vehicle-treated WT hippocampal slices, as revealed by an independent t-test (t(32) = 2.2, p = .04).

### DCS partially restores BDNF levels in brainstem and striatum of *Mecp2*^*tm1*.*1Jae/y*^ mice

**Fig 4:**
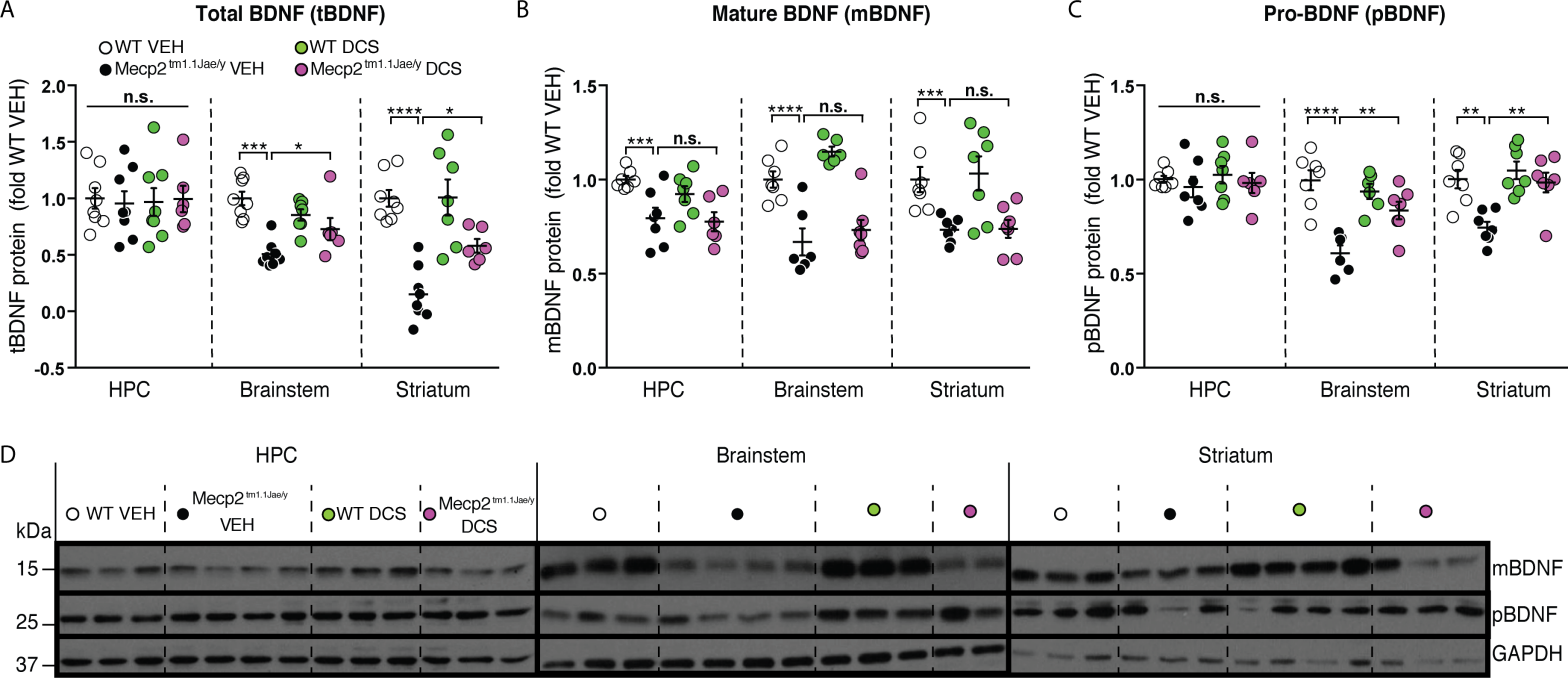
DCS (20 mg/kg/day IP) partially restores proBDNF (pBDNF) in the brainstem and striatum of *Mecp2*^*tm1*.*1Jae/y*^ mice. A. The brainstem and striatum but not hippocampus (HPC) had lower levels of total BDNF (tBDNF) protein. DCS had no effect on BDNF protein levels in any brain region in WT mice. DCS increased BDNF in the brainstem and striatum of *Mecp2*^*tm1*.*1Jae/y*^ mice. DCS had no effect on BDNF levels in the hippocampus. B. The HPC, brainstem and striatum had lower levels of mature BDNF (mBDNF) and DCS had no effect on protein levels in any of the brain regions. C. The brainstem and striatum but not HPC had lower levels of proBDNF (pBDNF) protein. DCS had no effect on pBDNF in any brain region in WT mice, however it increased pBDNF levels in the brainstem and striatum of *Mecp2*^*tm1*.*1Jae/y*^ mice. WT VEH n = 8, WT DCS n = 7–8, *Mecp2*^*tm1*.*1Jae/y*^ VEH n = 8, *Mecp2*^*tm1*.*1Jae/y*^ DCS n = 6. *p < .05, ***p < .001, ****p < .0001. D. Representative western blot images showing mBDNF, pBDNF and GAPDH in HPC, brainstem and striatum of all groups.

Brain-derived neurotrophic factor (BDNF) is a downstream target of MeCP2-mediated signaling [46,47], and reduced BDNF levels may contribute to the pathogenesis of RTT [48–50]. Stimulation of NMDA receptors increases release of neuronally-derived BDNF [51–54], and DCS treatment restores BDNF levels after neurologic injury in mice [55]. Accordingly, we wondered whether the improvement in behavior and synaptic transmission seen in *Mecp2*^*tm1*.*1Jae/y*^ mice after treatment with DCS might be associated with augmented BDNF levels in the brain. First using ELISA, we found that total BDNF (tBDNF) protein levels in *Mecp2*^*tm1*.*1Jae/y*^ mice were significantly lower in the brainstem (significant interaction, genotype x treatment, F_1,25_ = 14.04; p = 0.0067), and striatum (main effect of genotype F_1,25_ = 50.69; p < 0.0001; main effect of treatment, F_1,25_ = 5.97; p = 0.0425), compared to WT littermates (Fig 4A). DCS treatment significantly increased BDNF levels in both regions, without affecting levels in WT mice (Fig 4A). In the hippocampus, BDNF was unaltered in *Mecp2*^*tm1*.*1Jae/y*^ mice compared to WT littermates, and DCS-treatment had no effect in either genotype (Fig 4A). Next we examined levels of matureBDNF (mBDNF) and its precursor protein proBDNF (pBDNF) using isoform-specific antibodies. We found that mBDNF levels were significantly lower in the hippocampus (main effect of genotype F_1,23_ = 16.57; p = 0.0005), brainstem (main effect of genotype F_1,22_ = 52.91; p < 0.0001) and striatum (main effect of genotype F_1,24_ = 4.453; p < 0.0454) of *Mecp2*^*tm1*.*1Jae/y*^ mice compared to WT littermates. Surprisingly, DCS treatment had no effect in any of the brain regions (Fig 4B). By contrast, pBDNF was unaltered in the hippocampus but was significantly lower in the brainstem (significant interaction, genotype x treatment, F_1,22_ = 12.79; p = 0.0017) and striatum (significant interaction, genotype x treatment, F_1,24_ = 4.459; p = 0.0453). DCS treatment significantly increased pBDNF in both of these regions (Fig 4C).

## Conclusions

DCS, a cyclic analog of D-alanine, is already FDA-approved for use in relatively high doses as an antibiotic treatment for multiple drug-resistant strains of *M*. *tuberculosis* by virtue of its ability to inhibit crucial enzymes involved in peptidoglycan synthesis [56,57]. The dose of DCS administered here to mice (20 mg/kg) corresponds to 1.62 mg/kg for a human, based solely on body surface area conversion [58]. Assuming an average child mass of 32 kg, this would correspond to a daily dosing of 52 mg, which is much lower than the maximum recommended pediatric dose of 1 gram of DCS for active tuberculosis. Thus, there is hope that a traditional dose of 500 mg– 1 gram a day of DCS could have beneficial effects in humans with RTT. Notably, side effects of DCS are relatively mild and rare, and patients have reported that they are generally unaware of whether they have ingested DCS or placebo [59]. This, in addition to the wide availability and established safety of DCS, has recently helped facilitate human trials for other potential applications of this agent as well. As DCS also safely increases calcium influx by acting as a partial agonist at the glycine recognition site on the NRI subunit of the NMDA-subtype glutamate receptor [21], applications for CNS disorders have been sought. Given the role of NMDA receptors in learning, one of the most heavily emphasized potential CNS applications of DCS has thus far been the possibility of facilitating exposure therapy for people suffering from fear and anxiety disorders. Our data presented here now also suggest that treatment with DCS might help mitigate some aspects of the synaptic deficits and symptoms experienced by patients suffering from RTT as well. Of note, we observed a small but significant decrease in LTP in mice treated with DCS, and it will be important to monitor humans for side effects that may correlate with LTP, such as cognitive decline. Thus far, this has not been reported in the extensive clinical literature on DCS.

Previous work has shown that mice with mutations in *Mecp2* have significant cognitive impairments that may in part be mediated by long-term and short-term synaptic plasticity deficits [15,60,61]. In this study, we report significant decrements in hippocampal LTP, specifically in the Scaffer collateral pathway, as well as significant decreases in PPR in the *Mecp2*^*tm1*.*1Jae/y*^ mouse model of RTT. Importantly, we demonstrate that chronic treatment with DCS reverses the short-term synaptic deficits seen in PPR at 20, 30 and 50 sec interstimulus intervals, indicating that presynaptic function may be restored by long-term treatment with DCS. Nelson et al [16]demonstrated significant alterations in short-term plasticity in hippocampal cell cultures deficient in MeCP2. This may be the result of imbalances in excitatory/inhibitory transmission, as mEPSC frequency was substantially decreased in MeCP2 KO hippocampal cell cultures [16]. Treatment of *Mecp2*^*tm1*.*1Jae/y*^ mice with DCS may rectify this imbalance between excitatory and inhibitory tone, thus ameliorating the deficits in PPRs seen in *Mecp2*^*tm1*.*1Jae/y*^ mice. Of note, synaptic inhibition was not measured in this study, and follow-up work should focus on this aspect of physiology. Past experiments in other mouse models of autism and *Fragile X* syndrome [62–64] have indicated that restoration of excitatory and inhibitory imbalance may provide a therapeutic target for individuals suffering from these neurodevelopmental disorders. Indeed, recent work has demonstrated that administration of picrotoxin, a GABA_A_ antagonist, can reverse the synaptic impairments that arise as a function of MeCP2 overexpression [65]and may function to mitigate imbalances in excitatory/inhibitory neurotransmission.

DCS did not rescue the LTP deficits in the *Mecp2*^*tm1*.*1Jae/y*^ mice used in this study. The lack of improvement in LTP might be due to the fact that DCS binds to the glycine-binding site on the NR1 subunit of NMDA receptors and not the NR2 subunits, which presumably are necessary for supporting LTP [66,67]. Given that we observe no significant differences in BDNF expression in the hippocampus of *Mecp2*^*tm1*.*1Jae/y*^ mice compared to WT mice, the deficits in LTP in *Mecp2*^*tm1*.*1Jae/y*^ mice are not necessarily due to alterations in hippocampal BDNF expression. Indeed, *Mecp2*^*308/y*^ mice, another mouse model of RTT display deficient LTP with normal hippocampal *Bdnf* mRNA expression [60]. Thus, factors besides BDNF may be responsible for the deficits in hippocampal long-term synaptic plasticity observed in Mecp2 mutant mice. Other studies have indicated that DCS if given chronically may actually function as an NMDA antagonist, as prolonged exposure to DCS desensitizes the NMDA receptor complex [68,69]. Acute administration of DCS facilitates maze learning in mice, but chronic administration of DCS (15 days) significantly impairs consolidation and memory retrieval [70]. These data may explain the difference in LTP responses between the two WT groups. Future studies will need to focus on the mechanisms putatively involved in impairments in LTP in Mecp2 mutant mice.

The respiratory distress endured by patients with RTT is modeled in *Mecp2*^*tm1*.*1Jae/y*^ mice as an increase in breathing apneas [71–73], (and has been linked to an abnormally highly excited default state of the brainstem respiratory network [72–76]. A crucial part of this respiratory network is the nucleus of the solitary tract (nTS), where BDNF normally modulates excitability at primary afferent synapses by inhibiting post-synaptic responses to excitatory glutamatergic transmission [77]. It has previously been shown that brief exposure of *Mecp2*-deficient brainstem slices to BDNF reduces synaptic hyperexcitability in the nTS [78]. Furthermore, administration of LM22A-4, a small molecule non-peptide BDNF loop-domain mimetic [79], to a mouse model of RTT decreases synaptic hyperexcitability in the brainstem and normalizes breathing apneas [80,81]. Here, we have observed lower levels of BDNF in the brainstem of *Mecp2*^*tm1*.*1Jae/y*^ mice, and show that moderate mitigation of breathing apneas is associated with a DCS-mediated increase in brainstem proBDNF and not mature BDNF. We further observed deficient striatal mature BDNF and proBDNF in *Mecp2*^*tm1*.*1Jae/y*^ mice, with proBDNF and not mature BDNF also elevated by treatment with DCS, which could relate to the improved motor function. This is supported by a recent finding of an increase in striatal proBDNF and not mature BDNF in the 6-hydroxydopamine model of Parkinson’s disease with improvement in motor function following treatment with the neuroprotective compound, Diphenyl diselenide [82]. Indeed, this also appears consistent with previous findings that administration of fingolimod, a sphingosine-1 phosphate receptor modulator that increases total BDNF in the striatum of *Mecp2*^*tm1*.*1Jae/y*^ mice, improves locomotor activity in these mice as well [83]. It is intriguing that lower levels of proBDNF and not mature BDNF are elevated by DCS treatment in the brainstem and striatum, given that proBDNF is a negative regulator of synaptic plasticity [84], suggesting that yet unknown mechanisms could be attributed to the actions of proBDNF. Future studies will address the precise mechanism by which proBDNF may contribute to the improvement of irregular breathing and motor function. Additionally, it is hoped that future work in the field may focus on characterization of synapses in brainstem nuclei involved in the irregular breathing phenotype, as this is of paramount importance to patients with RTT.

Taken together, our data provide a novel therapeutic treatment for deficits in short-term synaptic plasticity in this mouse model of RTT. Future experiments will focus on developing and testing hippocampal-specific behavioral correlates of this deficit. Importantly, peripheral treatment with DCS also normalized BDNF expression in the brainstem, which may account for the improvement that we observe in sleep apneas. Given that we also see increases in BDNF expression in the thalamus and striatum, other behaviors may also be improved by DCS treatment. Importantly, since in RTT females are mostly affected, a test of the effects of DCS in female Mecp2 heterozygous mice is critical for determining the suitability of DCS as a possible treatment for patients. Future studies will focus on this as well as behaviors that may be markedly improved by DCS treatment, including striatal- and hippocampal-dependent behaviors, and also on evaluation of whether the beneficial effect of DCS is observed in additional preclinical models of RTT in other laboratories as well. If so, then clinical trials of DCS treatment of patients with RTT would be warranted. In addition, it is important to note that female RTT patients are a mosaic, and therefore mutated and phenotypically normal neurons and glial cells are intermixed in their brains. Thus, in future testing of the putative therapeutic efficacy of any drug for patients with RTT, including FDA-approved DCS, it will be essential to assess possible side effects on normal cells by rigorously assessing the behavioral effect in WT littermates as well.

## Supporting information

S1 ARRIVE Checklist (PDF)
