# Correction: D-cycloserine improves synaptic transmission in an animal mode of Rett syndrome

**DOI:** 10.1371/journal.pone.0192057

**Published:** 2018-01-25

**Authors:** Elisa S. Na, Héctor De Jesús-Cortés, Arlene Martinez-Rivera, Zeeba D. Kabir, Jieqi Wang, Vijayashree Ramesh, Yasemin Onder, Anjali M. Rajadhyaksha, Lisa M. Monteggia, Andrew A. Pieper

In the title of this article, the word “mode” should be “model.” The correct title is: “D-cycloserine improves synaptic transmission in an animal model of Rett syndrome.” The correct citation is: Na ES, De Jesús-Cortés H, Martinez-Rivera A, Kabir ZD, Wang J, Ramesh V, et al. (2017) D-cycloserine improves synaptic transmission in an animal model of Rett syndrome. PLoS ONE 12(8): e0183026. https://doi.org/10.1371/journal.pone.0183026
